# rDNA and mtDNA analysis for the identification of genetic characters in the hybrid grouper derived from hybridization of *Cromileptes altivelis* (female) × *Epinephelus lanceolatus* (male)

**DOI:** 10.1186/s12863-023-01188-5

**Published:** 2024-01-12

**Authors:** Liu Cao, Pan Chen, Xingrong Hou, Jun Ma, Ning Yang, Yan Lu, Hai Huang

**Affiliations:** 1 Yazhou Bay Innovation Institute, Sanya, 572022 China; 2 Key Laboratory of Utilization and Conservation for Tropical Marine Bioresources of Ministry of Education, Sanya, 572022 China; 3 Hainan Key Laboratory for Conservation and Utilization of Tropical Marine Fishery Resources, Sanya, 572022 China; 4 Hainan Tropical Ocean University, Sanya, 572022 China

**Keywords:** Hybridization, Genetic trait, rDNA evolution, DNA methylation, Grouper

## Abstract

**Background:**

Hybridization is a useful strategy to produce offspring with more desirable phenotypic characteristics than those of parents. The hybrid grouper derived from the cross of *Cromileptes altivelis* (♀, 2n = 48) with *Epinephelus lanceolatus* (♂, 2n = 48) exhibits improved growth compared with its female parent, which makes it valuable to aquaculture. However, the genetic traits of the hybrid grouper are poorly understood.

**Results:**

The observations showed that the hybrid grouper was diploid (2n = 48) and displayed intermediate morphology with the parent's measurable characteristics. The ribosomal DNA (rDNA) and mitochondria DNA (mtDNA) were characterized at molecular and phylogenetic level. High similarity and low genetic distance of 5S rDNA and mtDNA sequences between the hybrid grouper and *C. altivelis* showed that the hybrid grouper had a closer genetic relationship with female parents. The reconstructed phylogenetic tree based on COI gene and D-loop region of mtDNA recovered that mtDNA was maternally inherited in the hybrid grouper. Additionally, the DNA methylation level of 5S rDNA intergenic spacers (IGS) sequence was tested in here. The results showed that the DNA methylation status of the hybrid grouper was significantly lower than that of *C. altivelis*.

**Conclusion:**

Results of this study provide important data on the genetic characteristics of the hybrid derived from the cross of *C. altivelis* and *E. lanceolatus*, and contribute the knowledge of both evolution and marine fish breeding.

**Supplementary Information:**

The online version contains supplementary material available at 10.1186/s12863-023-01188-5.

## Introduction

*Cromileptes altivelis* (Serranidae: Cromileptes) and *Epinephelus lanceolatus* (Serranidae: Epinephelus) have been widely cultivated in China and Southeast Asian countries, possessing commercial importance fishing market species [1, 2]. The appearance of *C. altivelis* is distinct from fish of the subfamily Epinephelinae, because it has a concave slope on the back of head, hump-like bulge on the back and dark gray-brown body with black spots, so it can also be called “humpback grouper” [3]. Juvenile *C. altivelis* is kept as ornamental fish, while the adult is important to commercial mariculture. *E. lanceolatus* (also named as giant grouper) is regarded as a high-value species in fish markets due to its fast growing, large size and high nutritional value [4]. It is distinguished by small eye, wide interorbital area, body with irregular whitish blotches and numerous small black spots, and fins slightly yellowish with irregular blackish and whitish marking [5]. Groupers are usually considered to be protogynous hermaphrodites, and their sex type changes from female to male as they mature. Mature males are hard to obtain because of their huge body size and long time to maturity, so it is quite difficult to artificially reproduce them [6]. Hybridization is the most effective and widely used technique in the artificial breeding of grouper, which can produce offspring with more desirable phenotypic characteristics than those of the parent [7]. Many excellent offspring have been obtained using *C. altivelis* or *E. lanceolatus* as parent: *C. altivelis* (♀) × *E. tukula* (♂) [8], *E. moara* (♀) × *E. lanceolatus* (♂) [9], *E. coioides* (♀) × *E. lanceolatus* (♂) [10]. The hybrid grouper derived from the crossing of *C. altivelis* (♀) and *E. lanceolatus* (♂) exhibits the combined advantages of the parent species, with faster growth and improved taste when compared with wild groupers. However, the genetic traits in the hybrid grouper are poorly understood.

Morphological research relies heavily on statistics and classification methods using measurable, countable, and partly descriptive traits in fish [11]. Where parents differ physically from one another, morphological analysis is a useful tool in revealing the genetic relationship between hybrid progenies and parents [10]. However, it cannot effectively identify the genetic relationship between offspring and parents only by phenotypic characteristics. When combined with morphological analysis, molecular markers can greatly improve the accuracy of hybrid identification. In eukaryotes, the ribosomal DNA multigene family is organized in coding regions and non-transcribed spacer (NTS) regions. Because concerted and birth-and-death evolution mechanisms act simultaneously in rDNA, the coding regions show high conservation, while the NTS regions exhibit different rates of variation within and between taxa. This trait of rDNA sequence structure makes it a good molecular marker for the investigation of rapid evolutionary events [12–14]. Intergenic spacers (IGS) can separate these rDNA repeats, and present between two successive genes [15]. IGS transcripts play an important role in the epigenetic control of the rDNA locus [16]. DNA methylation, essentially the methylation of cytosine nucleotides, is the first identified epigenetic mechanism [17] and has been extensively studied. Accumulated studies suggested that DNA methylation was closely correlated with the heterosis in animals. Ou et al*.* have reported that the association of DNA methylation with the growth heterosis in snakehead fish [18]. Jiang et al*.* have found that DNA methylation can be involved in the heterosis formation in pig hybrids [19]. In the Pacific *oyster Crassostrea gigas*, the DNA methylation level is associated with the superior growth of the hybrid crosses [20].

Mitochondria DNA (mtDNA) is also valued for tracking the ancestry of breeds back hundreds of generations [21], because the organization in most fish mtDNA genomes is quite conserved [22]. Many researchers analyzed the mtDNA D-loop region and cytochrome oxidase subunit 1 (COI) to assess phylogenetic relationships and maternal origin of different fish populations [23–25]. In this study, we investigated the phenotypic characteristics, chromosomal numbers, rDNA and mtDNA sequences, the DNA methylation level of 5S rDNA IGS sequences between the hybrid grouper and its parents. Based on our data, the genetic traits of the hybrid grouper (*C. altivelis* (♀) × *E. lanceolatus* (♂)) were explored.

## Materials and methods

### Ethics statement

All experiments were conducted in accordance with the guidelines statement of the Administration of Affairs Concerning Animal Experimentation of China. The health of the fish and environmental conditions were monitored daily.

### Collection of experimental fish

The fry of *E. lanceolatus* were derived from Delin Chengxin Aquaculture Co., Ltd, Hainan Province, China. Two-month-old *C. altivelis* and two-month-old the hybrid groupers were collected from Hainan Chenhai Aquatic Co., Ltd, Hainan Province. All samples were raised up to two years old at Delin Chengxin Aquaculture Co., Ltd. Total genomic DNA was isolated from peripheral blood cells.

### Measurement of morphological data

A total of 30 individuals aged 2 years of each from *C. altivelis*, *E. lanceolatus* and the hybrid grouper were selected at random. Fish were anaesthetized with 3-aminobenzoic acid ethyl ester methanesulfonate before measuring. The total length, standard length, body height, head length, caudal peduncle length and caudal peduncle height were measured. The average ratios of total length to standard length, head length to standard length, body height to standard length, caudal peduncle length to standard length and caudal peduncle height to standard length were recorded.

### Preparation of chromosome spreads

The fin cells of *C. altivelis*, *E. lanceolatus* and the hybrid grouper were used for chromosome preparation. The method of fin cell culture and chromosome preparation described by Alvarez et al. was used with some modification [26]. Before collecting cells, concanavalin was added to the cells three times in one-hour intervals. The final concentration was 0.1 μg/mL. The collected cells underwent hypotonic treatment with 0.075 mol/L KCl at 37 °C for 15–20 min and were then fixed in methanol-acetic acid (3:1) for 20 min with two changes. Chromosome preparations were examined under an oil lens. Two-hundred metaphase spreads were examined for each fish sample.

### Analysis of the genetic traits of rDNA and mtDNA

Thirty *C. altivelis*, thirty *E. lanceolatus* and fifty the hybrid grouper were selected at random. Genomic DNA was extracted from the blood and fin tissue. All PCR primers were listed in Table S1. The PCR amplification program was conducted for 30 cycles with an annealing temperature of 30 s at 57 °C for 5S rDNA, 35 s at 55 °C for COI gene and 30 s at 50 °C for D-loop region. All PCR products underwent a series of applications such as purification, cloning, and sequencing. Multiple sequence alignment analysis was conducted using BioEdit and Clustal W [27, 28]. The RepeatMasker program (http://www.repeatmasker.org/) was used to search for 5S gene repeated elements inside spacers. All sequences were submitted to the NCBI.

### Methylation analysis of 5S rDNA IGS sequence

The DNA methylation level in 1021 bp upstream sequence of coding region was tested and analyzed to investigate the DNA methylation patterns of IGS regions in the hybrid grouper and its parents (Supplementary Figure 1). Muscle tissues were applied to extract genomic DNA in *C. altivelis* (five individuals), *E. lanceolatus* (seven individuals) and the hybrid grouper (five individuals). MethPrimer software package (http://www.urogene.org/methprimer/index.html) was applied for designing PCR primers (Table S1). PCR amplification was conducted with an annealing temperature of 30 s at 50 ~ 60 °C by KAPA HiFi HotStart Uracil + ReadyMix PCR Kit (Kapa Biosystems, Wilmington, MA, USA). Steps of bisulfite modification and pyrosequencing were showed as follow: firstly, genomic DNA (1 μg) was converted (by the ZYMO EZ DNA Methylation-Gold Kit (Zymo Research, Irvine, CA, USA)) and one-tenth of the elution products were used as templates. Bisulfite sequencing PCR products of IGS sequences were pooled equally, 5'-phosphorylated, 3' -dA-tailed and ligated to barcoded adapter using T4 DNA ligase (NEB). Secondly, Barcoded libraries from all samples were sequenced on Illumina platform [29]. Thirdly, the raw reads needed to remove adapters and filter the low-quality sequences (Trimmomatic-0.36). Finally, clear reads were obtained. The methylation levels of individual cytosines were calculated as the ratio of the total number of methylated CpG cytosines to the number of sequenced clones. A two-tailed Fisher’s Exact Test was used to test the methylated and unmethylated counts for each cytosine between two groups [30].

### Phylogenetic analysis and genetic distances

Mitochondrial genome sequences were obtained from the NCBI GenBank. All accession numbers of the sequences were listed in Table S2. Phylogenetic trees were constructed by a maximum-likelihood approach (ML) in the program PhyML 3.0 (http://www.atgc-montpellier.fr/phyml/) and Bayesian inference (BI) using MrBayes 3.1.2 (http://mrbayes.sourceforge.net/). A generalized time reversible (GTR) model with a proportion of invariable sites (I) and a gamma distribution (G) was selected for the concatenated dataset in ML. Nodal robustness was estimated by bootstrap percentage values (BP). The BP values of nodes of the subtree were obtained after 1000 replicates. MrBayes used Markov chain Monte Carlo (MCMC) methods to estimate the posterior distribution of model parameters. Convergence occurred when average standard deviation of split frequencies fell below 0.01. Posterior probability (PP) values were applied to estimate branch support. The phylogenetic trees were shown using the Figtree program v.1.4.4 (http://tree.bio.ed.ac.uk/software/figtree/).

Between-group mean distances were calculated using MEGA 7 with a p-distance model.

## Results

### Morphological traits

**Fig. 1:**
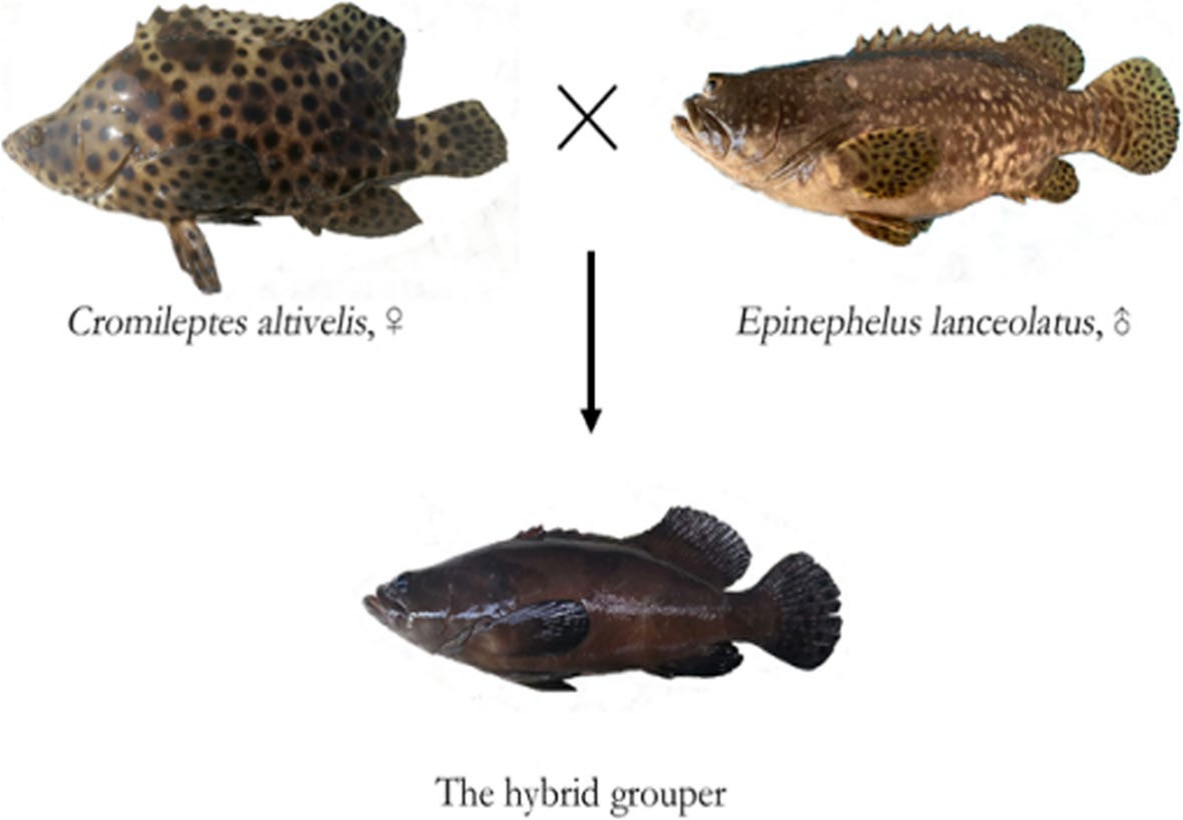
The appearances of *Cromileptes altivelis, Epinephelus lanceolatus* and the hybrid grouper

There were obvious phenotypic differences between the hybrid grouper and its parents (*C. altivelis* and *E. lanceolatus*) (Fig. 1). Regarding body color, *C. altivelis* is white with scattered black dots and *E. lanceolatus* is dark brown with scattered black spots. The hybrid grouper is dark brown without any obvious black spots, which differed visibly from both parents.

The measurable traits in *C. altivelis*, *E. lanceolatus,* and the hybrid grouper are presented in Table 1. The values of total length, standard length, head length, body height, and caudal peduncle height for *E. lanceolatus* were greater than in *C. altivelis*. However, the caudal peduncle length was greater for *C. altivelis* than for *E. lanceolatus.* All measurable traits differed significantly between *E. lanceolatus* and *C. altivelis* (*p* < 0.05)*.* For the hybrid grouper, all measurable traits had values higher than those of *C. altivelis* and lower than those of *E. lanceolatus* (excepting for caudal peduncle length). All measurable traits with the exception of head length and body height showed significant differences between the hybrid and *C. altivelis* (*p* < 0.05)*.*

**Table 1:** Comparison of the measurable traits between *Cromileptes altivelis*, *Epinephelus lanceolatus* and the hybrid grouper

measurable traits	*Cromileptes altivelis*	*Epinephelus lanceolatus*	The hybrid grouper
Total length (cm)	33.07 ± 2.19^a,b^	43.03 ± 3.20	36.51 ± 1.78
Standard length (cm)	27.55 ± 1.87^a,b^	35.80 ± 2.93	30.32 ± 2.08
Head length (cm)	11.09 ± 0.81^a^	14.43 ± 1.08	11.24 ± 0.25
Caudal peduncle length (cm)	2.96 ± 0.28^a,b^	2.65 ± 0.48	3.18 ± 0.30
Body height (cm)	9.52 ± 0.91^a^	12.92 ± 1.42	9.81 ± 0.60
Caudal peduncle height (cm)	3.11 ± 0.20^a,b^	4.65 ± 0.40	3.37 ± 0.24
Total length/ Standard length	1.20 ± 0.01	1.20 ± 0.01	1.21 ± 0.03
Head length/ Standard length	0.40 ± 0.01	0.40 ± 0.02	0.37 ± 0.03
Body height/ Standard length	2.90 ± 0.14	2.78 ± 0.16	3.10 ± 0.21
Caudal peduncle length/ Standard length	0.11 ± 0.01	0.07 ± 0.01	0.10 ± 0.01
Caudal peduncle height/ Standard length	0.11 ± 0.01	0.13 ± 0.01	0.11 ± 0.01

^a^ there is a significant difference between *Cromileptes altivelis* and *Epinephelus lanceolatus* (*p* < 0.05)

^b^ there is a significant difference between *Cromileptes altivelis* and the hybrid grouper (*p* < 0.05)

To control for differences in growth rate between species, the ratios of the measurable traits were recorded in the hybrid grouper and its parents. The ratios of measurable traits did not differ significantly between the hybrid grouper, *C. altivelis,* and *E. lanceolatus* (*p* > 0.05), which indicates that all three groupers show similar morphological characteristics in terms of total length, head length, standard length, caudal peduncle length, body height, and caudal peduncle height.

### Examination of chromosome number

**Fig. 2:**
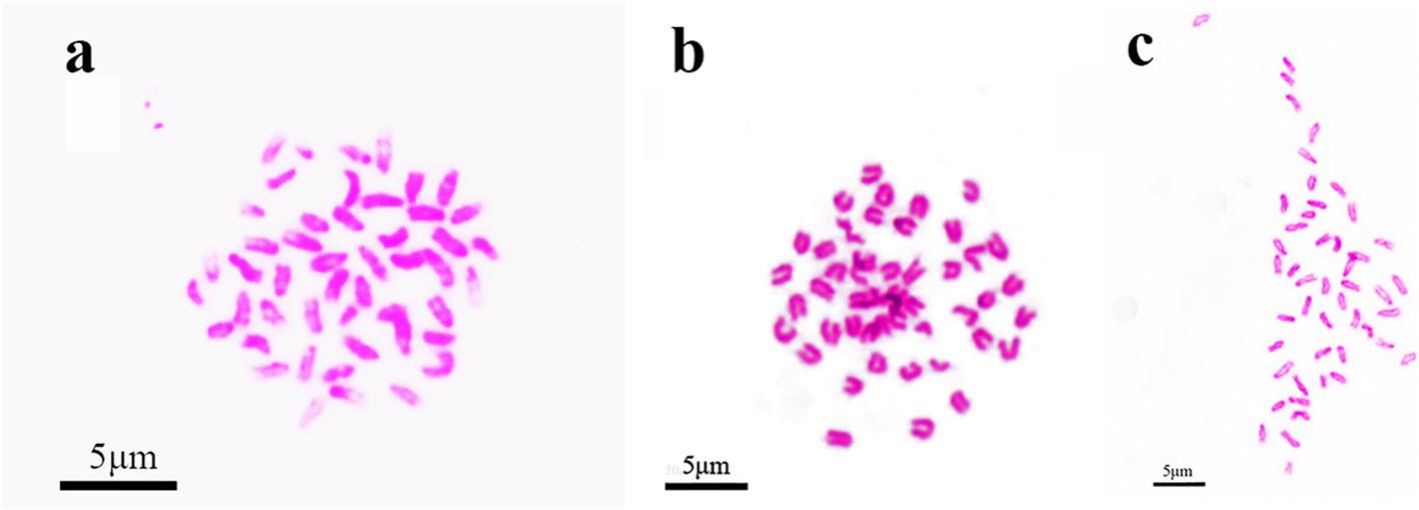
Chromosome spreads at metaphase in *Cromileptes altivelis, Epinephelus lanceolatus* and the hybrid grouper. **a**. The 48 chromosomes of *Cromileptes altivelis*. **b**. The 48 chromosomes of *Epinephelus lanceolatus*. **c**. The 48 chromosomes of the hybrid grouper

The distribution of chromosome numbers in *C. altivelis*, *E. lanceolatus*, and the hybrid grouper is illustrated in Fig. 2. Among *C. altivelis*, 92% of the chromosomal metaphases had 48 chromosomes. Among *E. lanceolatus*, 90% of the chromosomal metaphases had 48 chromosomes. Among the hybrid grouper, 89% of the chromosomal metaphases had 48 chromosomes.

### Sequence organization and analysis of rDNA and mtDNA

**Fig. 3:**
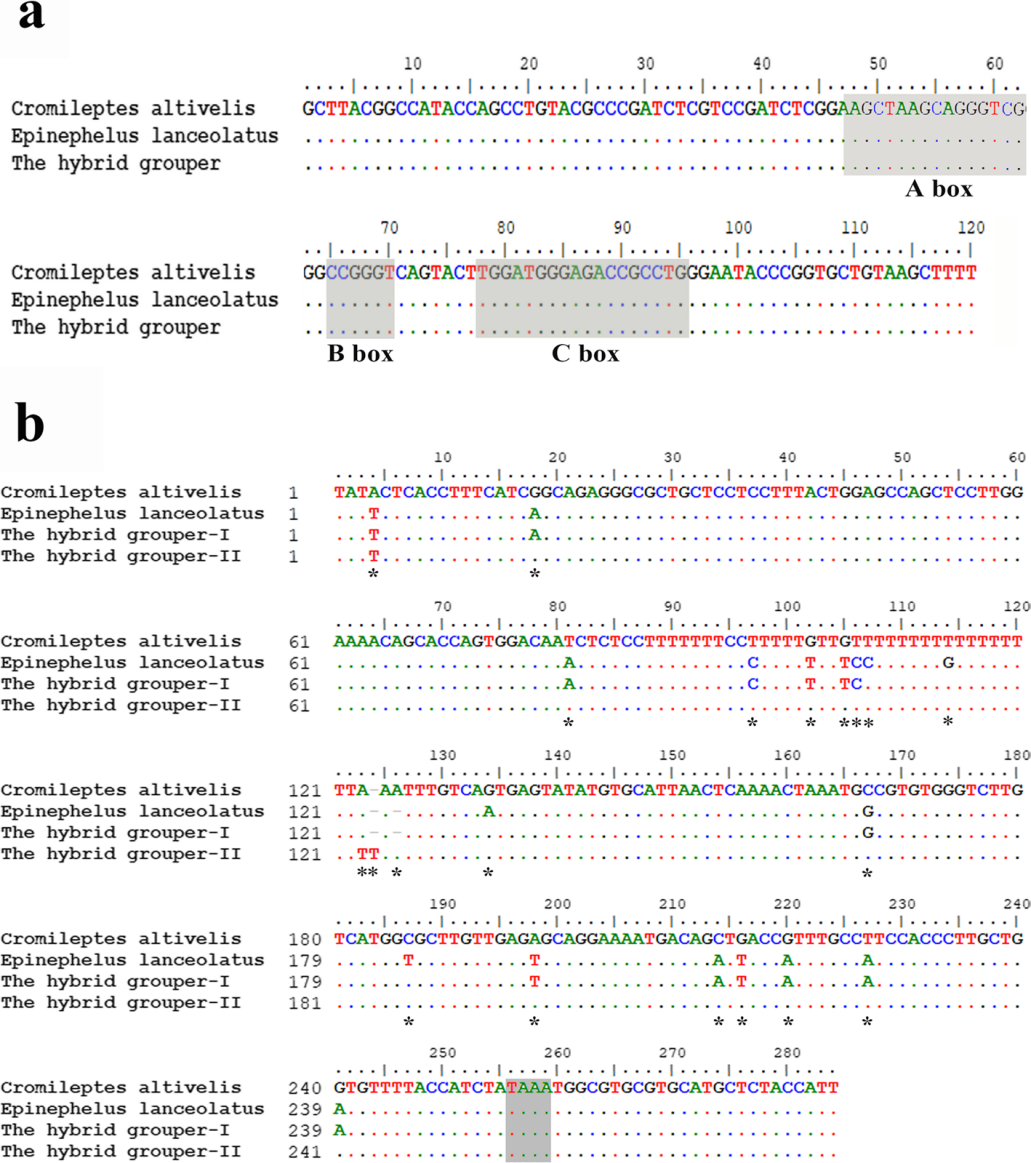
Representative sequences of 5S rDNA. **a**. Complete 5S coding regions from *Cromileptes altivelis, Epinephelus lanceolatus* and the hybrid grouper. Internal control regions of the coding region are shaded. **b**. Comparison of the NTS sequences from *Cromileptes altivelis, Epinephelus lanceolatus* and the hybrid grouper, the NTS upstream TATA elements are shaded; asterisks mark variable sites in the NTS

**Fig. 4:**
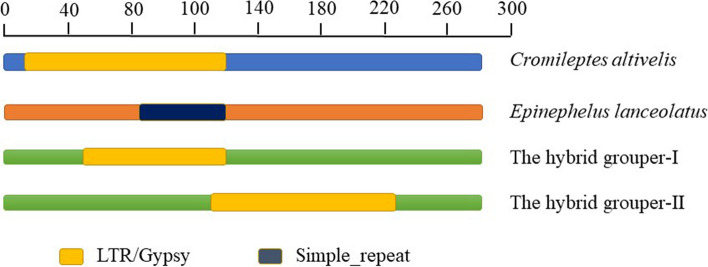
Schematic representation of the 5S rDNA sequences showing the different types of elements found in the NTS of *Cromileptes altivelis, Epinephelus lanceolatus* and the hybrid grouper

A single band of 5S rDNA was observed in the genomes of *C. altivelis* (403 bp, accession numbers: OM289959), *E. lanceolatus* (402 bp, accession numbers: OM289957) and the hybrid grouper. Based on the nucleotide compositions of NTS, 5S sequences in the hybrid grouper were divided into two categories (type I: 402 bp, accession numbers: OM289958; type II: 404 bp, accession numbers: OP244353). All 5S clones in samples had the same coding region sequences (Fig. 3a). The internal control regions (ICRs, i.e., the promoters for transcription)-Box A (positions 48–62), Box B (positions 65–70), and Box C (positions 78–95)-were detected in the 5S coding region. In 5S NTS region, several base substitutions or insertions/deletions were found in the hybrids and their parents. Sequence alignments of the NTS region with BLASTn showed 96.2% similarity between the hybrid grouper (type I) and *C. altivelis,* and 99.0% similarity between the hybrid grouper (type I) and *E. lanceolatus.* Type II of the hybrid grouper displayed higher conservation with *C. altivelis* (99.2%) compared with *E. lanceolatus* (95.0%) (Table 2). The TATA control element that regulates 5S gene transcription was generally located from -25 to -30 from the 5S, was also identifiable in the hybrid and parents (at -29 in all NTS sequences, where it was modified to TAAA) (Fig. 3b). In addition, long terminal repeat (LTR) retrotransposons were identified by the RepeatMasker program in the NTS regions of *C. altivelis* (pos. 18–122) and the hybrid grouper (type I: pos. 51–122; type II: pos. 117–223), and the (T)n simple repeat was identified in that of *E. lanceolatus* (pos. 88–122) (Fig. 4).

**Table 2:** The similarity and genetic distance between *Cromileptes altivelis*, *Epinephelus lanceolatus* and the hybrid grouper in 5S rDNA

Genetic distance Similarity	*Cromileptes altivelis*	*Epinephelu lanceolatus*	The hybrid grouper (Type I)	The hybrid grouper (Type II)
*Cromileptes altivelis*		0.064	0.050	0.007
*Epinephelu lanceolatus*	0.952		0.014	0.057
The hybrid grouper (Type I)	0.962	0.990		0.043
The hybrid grouper (Type II)	0.992	0.95	0.96

The length of COI gene sequences was 1545 bp in *C. altivelis*, *E. lanceolatus*, and the hybrid grouper (Supplementary Figure 2). Sequence alignments with BLASTn showed that the COI gene sequences of *C. altivelis* and *E. lanceolatus* in this study was identical with the sequences in NCBI database (*Cromileptes altivelis*: NC_021614.1, *Epinephelus lanceolatus*: KM386619.1). The similarity between the hybrid grouper and *C. altivelis* was 99.8% (Table 3). Partial D-loop region sequences (-800 bp) were obtained in *C. altivelis*, *E. lanceolatus*, and the hybrid grouper (Supplementary Figure 3). The similarity between the hybrid grouper and *C. altivelis* was 99.0% (Table 3).

**Table 3:** The similarity and genetic distance between *Cromileptes altivelis*, *Epinephelus lanceolatus* and the hybrid grouper in COI gene and D-loop region

	Genetic distance Similarity	*Cromileptes altivelis*	*Epinephelu lanceolatus*	The hybrid grouper
COI gene	*Cromileptes altivelis*		0.122	0.002
	*Epinephelu lanceolatus*	0.878		0.124
	The hybrid grouper	0.998	0.876	
D-loop region	*Cromileptes altivelis*		0.370	0.010
	*Epinephelu lanceolatus*	0.649		0.369
	The hybrid grouper	0.99	0.645	

### DNA methylation analysis of 5S rDNA IGS sequence

**Fig. 5:**
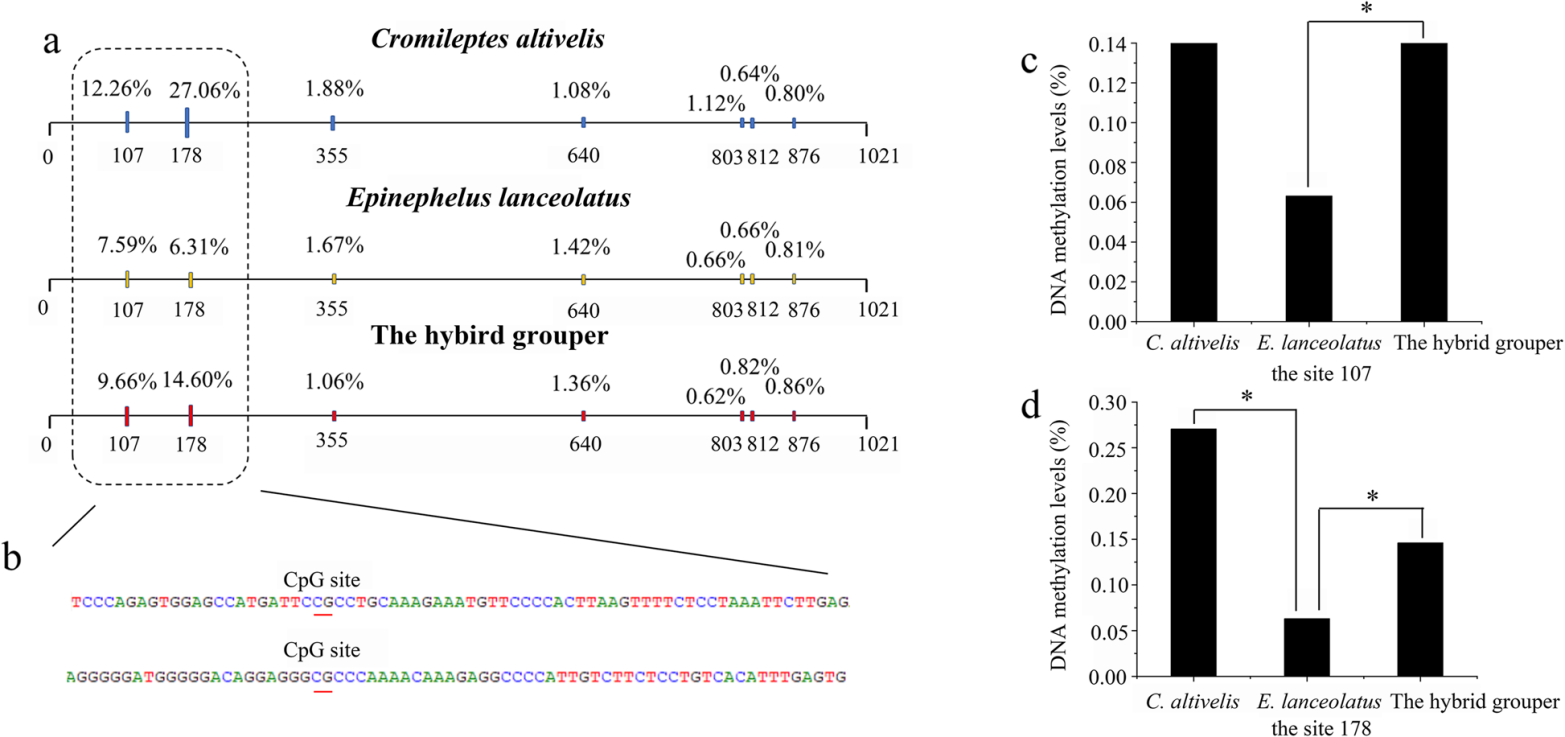
DNA methylation of 5S rDNA intergenic spacers (IGS) sequence in *Cromileptes altivelis*, *Epinephelus lanceolatus*, the hybrid grouper. **a**. Schematic representation of the DNA methylation level in CpG sites. The box indicates methylation level of CpG sites with significant difference between the hybrid grouper and parents. **b**. The partial sequence of IGS. The red lines indicate CpG sites. **c**. The DNA methylation level in site 107. **d**. The DNA methylation level in site 178. The symbol * indicates significant differences

Seven CpG sites were found in the 1021 bp upstream sequence of coding region between the hybrid grouper and its parents (Fig. 5). Only two CpG sites (the position of 107 and 178 in the sequence) exhibited the significant differences between the hybrid grouper and its parents (Fig. 5A, B). In site 107, the DNA methylation level of the hybrid grouper (9.66%) was higher than that of *E. lanceolatus* (7.59%). There was no significant difference between the hybrid grouper and *C. altivelis* (Fig. 5C). In site 178, the DNA methylation level of the hybrid grouper (14.60%) was higher than that of *E. lanceolatus* (6.31%), but lower than that of *C. altivelis* (27.06%) (Fig. 5D).

### Genetic distances and phylogenetic analysis

Genetic distances were estimated between *C. altivelis, E. lanceolatus* and the hybrid grouper in mtDNA and rDNA (Tables 2 and 3). The hybrids and *C. altivelis* had the minimal genetic distance (COI gene: 0.002; D-loop region: 0.010). In 5S rDNA, the genetic distance between the hybrids and *C. altivelis* ranged from 0.007 to 0.050 (average value = 0.0285) and was less than the distance between the hybrids and *E. lanceolatus*, which ranged from 0.014 to 0.057 (average value = 0.0355).

**Fig. 6:**
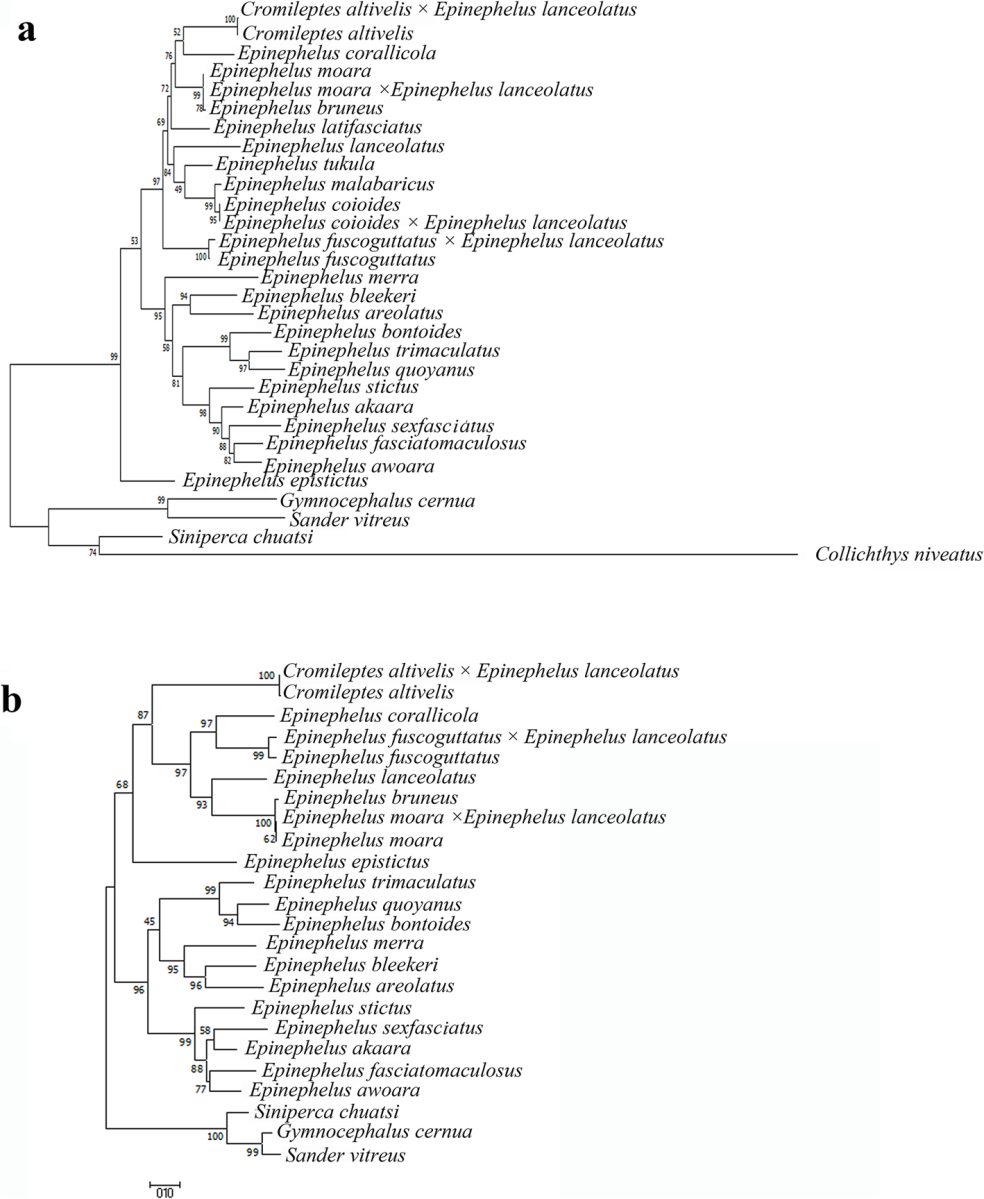
Maximum likelihood phylogenetic tree reconstructed based on COI gene (**a**) and D-loop region (**b**)

**Fig. 7:**
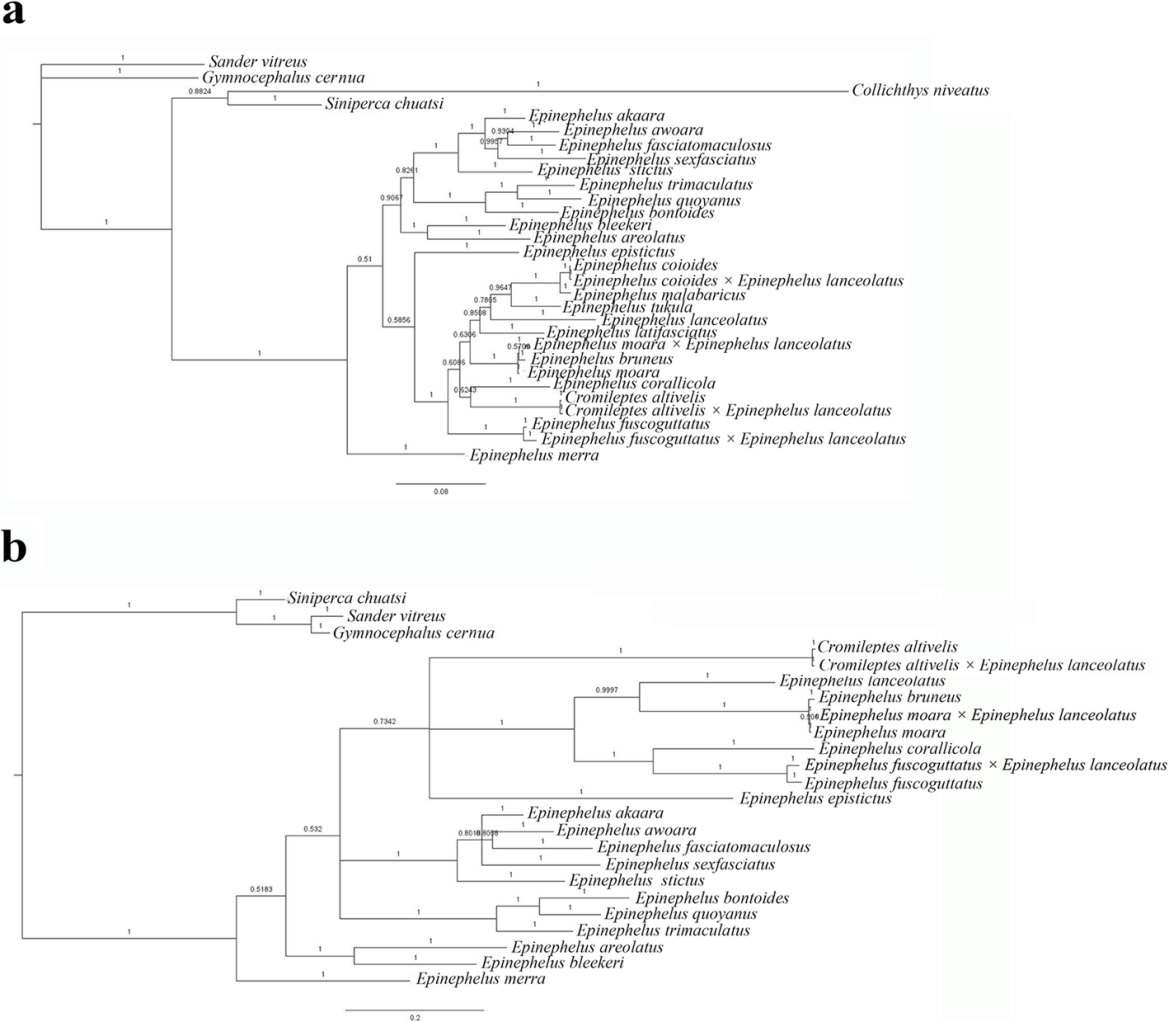
Bayesian phylogenetic tree reconstructed based on COI gene (**a**) and D-loop region (**b**)

Phylogenetic analyses generated similar tree topologies in ML (Fig. 6) and BI (Fig. 7). In the phylogenetic tree, the hybrid grouper and *C. altivelis* were closely clustered together (COI gene: BP = 100%, PP = 1; D-loop region: BP = 100%, PP = 1). *E. lanceolatus* and other Epinephelus species were clustered together.

## Discussion

Geometric morphometrics is considered to be one of the most powerful tools in fish shape analysis [31, 32], and can most intuitively express germplasm characteristics. In our results, obvious differences in the body color and blot shape were visible between the hybrid offspring and its parents (*Cromileptes altivelis* and *Epinephelus lanceolatus*) at 2 years of age. The hybrid grouper displayed intermediate morphology compared with the parents’ measurable characteristics. In total length, standard length, caudal peduncle length, and caudal peduncle height, there were significant differences between the offspring and *C. altivelis.* These observations indicated that the offspring's phenotypic traits exhibited the hybridity, and supported the viewpoint that the hybrid offspring exhibits faster growth than its female parent (*C. altivelis*) [33].

The chromosome number determines the characteristics of the species, and alterations in the chromosome number can reflect changes in genetic material during species evolution. Previous studies have shown that the ploidy level of *C. altivelis* and *E. lanceolatus* is diploid (2n = 48, [34, 35]). The ploidy level of the hybrid grouper has not been reported yet. In the present study, the hybrid grouper (2n = 48) was confirmed to be diploid by chromosome count. Such diploid offspring have previously been observed in other crosses between members of Epinephelinae: (*Epinephelus fuscoguttatus*, ♀ × *Epinephelus lanceolatus*, ♂), (*Epinephelus moara*, ♀ × *Epinephelus lanceolatus*, ♂), and *Epinephelus awoara*, ♀ × *Epinephelus tukula*, ♂) [35, 36].

To further analyze genetic characteristics, rDNA and mtDNA sequences were compared between the hybrid grouper and its parents. In rDNA coding region sequences, a low intra-specific and a high rate of homogenization were observed between the hybrid grouper, the female parent (*C. altivelis*), and the male parent (*E. lanceolatus*), in which the sequence similarity of 5S was 100%, indicating the presence of concerted evolution in the rDNA of these species. In the concerted evolution model, all the members of a multi-gene family evolve in concert. Variation in the repeat unit extends to all the member genes through gene conversion and unequal crossing over, which leads to homogenization in the units of the multigene family [37, 38].

Different 5S rDNA types have been reported in freshwater fish and several plants [39–42]. In the bony fish, there are often two 5S gene fragments [43], such as in *Carassius auratus* (2n = 100) and *Cyprinus carpio* (2n = 100). In our study, only one 5S gene band was found in the hybrid (2n = 48), *C. altivelis* (2n = 48), and *E. lanceolatus* (2n = 48). This result was previously observed in the genome of *Megalobrama amblycephala* (2n = 48)*.* Researchers [38] have proposed that the genomes of diploid *Carassius auratus* and *Cyprinus carpio* with 100 chromosomes likely experienced a polyploidization process and are ancient polyploidy fish. During the process, the variations and reorganizations of the genomes may have resulted in the appearance of new 5S rDNA. There was no evidence to suggest that *C. altivelis* and *E. lanceolatus* with 48 chromosomes went through the polyploidization process, and it seems reasonable to observe only one 5S gene type in their genome. In this study, all 5S gene sequences contained essential internal control regions (Box A, Box B, Box C, and TATA control element) for correct gene expression; thus, these 5S genes were likely to correspond to functional genes [42, 44].

In the 5S NTS region, nucleotide variations (including base substitutions or insertions/deletions) discovered in the hybrids, *C. altivelis* and *E. lanceolatus* demonstrate the divergence exerted in this region, as predicated by a birth-and-death evolution model [45]. Birth-and-death evolution leads to heterogeneity and pseudogenes in an rDNA multigene family [46, 47]. A mixed-mediated model between concerted evolution and birth-and-death has been described for the fish species *Diplodus sargus *[48], and *Halobatrachus didactylus *[49]. LTR retrotransposons were identified in the NTS regions of *C. altivelis* and the hybrid grouper. The presence of LTR retrotransposons has been associated with genome expansion because it can potentially give rise to numerous daughter copies [50].

Phylogenetic analysis revealed that all fish species were well clustered according to their taxonomic level. Matrilineal inheritance of mtDNA was observed in *C. altivelis* × *E. lanceolatus* and other hybrids (*E. fuscoguttatus* × *E. lanceolatus*, *E. moara* × *E. lanceolatus* and *E. coioides* × *E. lanceolatus*). All hybrids were closely clustered with their female parent. The genetic distance between the hybrid grouper and female parent (*C. altivelis)* was lower than those between the hybrid grouper and male parent (*E. lanceolatus*) in 5S rDNA and mtDNA. These results indicate that the hybrid grouper had a closer genetic relationship with female parent. The genetic distance of 5S rDNA between type I of the hybrids and type II of the hybrids was 0.043. Type I and *E. lanceolatus* had the minimum genetic distance (0.014), type II and *C. altivelis* had the minimum genetic distance (0.007). These observations have suggested the presence of genetic material from both parents in the hybrid grouper.

The synthesis of rRNA genes is closely related to the complex process of ribosome biogenesis, and can be regulated by changes of the transcription rate and the number of gene copies that are transcribed [51]. DNA methylation of rRNA gene promoter is an epigenetic switch, which can regulate rRNA transcription by controlling the number of rRNA genes in the on or off state [52]. It is widely known that hypomethylation of rRNA genes promoter can facilitates the binding of transcription factor on chromatin templates and drive the synthesis of rRNA in order for ribosomes biogenesis [51, 53]. In our results, methylation level of sites 107 and 178 presented the significant difference between the hybrid grouper and its parents. Among them, the methylation level of the hybrid grouper was in the middle compared with that of parents*.* Hybridization often results in dramatic genome reconfigurations including epigenetic changes [54]. Scholars have proposed that genome epigenetic regulation is an important factor for the formation of heterosis [55, 56]. In this study, results of geometric morphometrics indicated that the growth rate of 2-year-old the hybrid grouper was higher than that of *C. altivelis*, exhibiting the growth advantage. As it is known, grouper generally reaches sexual maturity at 3—5 years. Before reaching sexual maturity, the demand for energy is mainly used for growth and development. The protein synthesis is indispensable in the process of growth and development. Therefore, we infer that DNA methylation of 5S rDNA IGS sequences may indirectly affect the growth and development of grouper by regulating the rate of protein synthesis, but it still requires extensive evidences to support this viewpoint.

## Conclusions

The work in this study firstly confirmed the existence of diploid the hybrid grouper with 48 chromosomes. The molecular and phylogenetic analysis of rDNA and mtDNA recovered that the hybrid grouper has inherited the genetic materials from both of parents. The morphological trait data showed the hybrid grouper exhibits improved growth compared with *C. altivelis*. The DNA methylation of 5S rDNA IGS sequences in the hybrid grouper was also lower than that of *C. altivelis,* which provided a clue to understanding the growth advantage in the hybrid grouper.

### Supplementary Information

**Additional file 1:** **Table S1.** The primers used for PCR and BSP-PCR. **Table S2.** GenBank accession numbers for mitochondrial genome sequences.

**Additional file 2:** **Supplementary Figure 1.** Representative sequences of 5S rDNA intergenic spacers (IGS) sequence.

**Additional file 3:** **Supplementary Figure 2.** Representative sequences of COI gene from *Cromileptes altivelis*, *Epinephelus lanceolatus*, the hybrid grouper.

**Additional file 4:** **Supplementary Figure 3.** Representative partial sequences of D-loop region from *Cromileptes altivelis*, *Epinephelus lanceolatus*, the hybrid grouper.

## Data Availability

All raw data of bisulfite sequencing in *Cromileptes altivelis* × *Epinephelus lanceolatus* and parents have been uploaded to GenBank database (BioProject ID: PRJNA1009716, https://www.ncbi.nlm.nih.gov/bioproject/PRJNA1009716/). All 5S rDNA sequences generated during the current study are available in the NCBI (accession number: OM289957-OM289959, OP244353).
