# Supplementary material for: rDNA and mtDNA analysis for the identification of genetic characters in the hybrid grouper derived from hybridization of Cromileptes altivelis (female) × Epinephelus lanceolatus (male)
Source: BMC Genom Data. 2024 Jan 12;25:5. doi: 10.1186/s12863-023-01188-5 (PMC10787421; doi:10.1186/s12863-023-01188-5)
Supplement: Supplementary file 1 — Additional file 1: Table S1. The primers used for PCR and BSP-PCR. Table S2. GenBank accession numbers for mitochondrial genome sequences. [file 12863_2023_1188_MOESM1_ESM.docx]

Table S1 The primers used for PCR and BSP-PCR

|  | Sequences (5’-3’) | Usage |
| --- | --- | --- |
| 5S | F: TACGCCCGATCTCGTCCGATC | Cloning the 5S rDNA sequences |
|  | R: CAGGCTGGTATGGCCGTAAGC |  |
| COI | F: ATTACACGTTGATTTTTCTCGA | Cloning the COI sequences |
|  | R: TTAGSRTGRTYGAATTTGAAC |  |
| D-loop | F: TACTTCAAAGAGAGGAGATT | Cloning the D-loop sequences |
|  | R: AAATTWAAWAKTTACATTAAT |  |
| Primer 1 | F: AATGYGGTGGAGGTTATATGGGGTAA | Bisulfite sequencing |
|  | R: CAAATTAAACTCTCCCACTCAAATATAAC |  |
| Primer 2 | F: TTATAGGTAAATTTTGATTTYGATAAAAATTGAGATTG | Bisulfite sequencing |
|  | R: CCTTCATTCTACACAATAAAAATAAACTTTATTAATC |  |
| Primer 3 | F: GATGGGYGGTGGTTATTGAGTAATTTTATATTATTG | Bisulfite sequencing |
|  | R: CCTACTTTCACAATTCACTAAACTTTTTC |  |
| Primer 4 | F: AAAYGYGGAAGTTATAATTAAGTTGAGATTATG | Bisulfite sequencing |
|  | R: ACAACTTCTCCTCACTTCACAACTCC |  |
| Primer 5 | F: GGAGTTYGTTTGTTTYGTTAGAAGTTG | Bisulfite sequencing |
|  | R: CTATTTTCCAAAAAACTAACTCCAACAA |  |

Table S2 GenBank accession numbers for mitochondrial genome sequences

| Fish | | Accession number |
| --- | --- | --- |
| *Epinephelus akaara* | | KM458971 |
| *Epinephelus awoara* | | KT240121 |
| *Epinephelus fasciatomaculosus* | | NC_020782 |
| *Epinephelus sexfasciatus* | | NC_021765 |
| *Epinephelus stictus* | | NC_021133 |
| *Epinephelus trimaculatus* | | NC_021612 |
| *Epinephelus quoyanus* | | NC_021450 |
| *Epinephelus bontoides* | | NC_028428 |
| *Epinephelus bleekeri* | | NC_022848 |
| *Epinephelus areolatus* | | NC_020785 |
| *Epinephelus merra* | | NC_022509 |
| *Epinephelus epistictus* | | NC_021462 |
| *Epinephelus coioides* | | KM377093 |
| *Epinephelus coioides* × *Epinephelus lanceolatus* | | KP257572 |
| *Epinephelus malabaricus* | | NC_028406 |
| *Epinephelus tukula* | | NC_024039 |
| *Epinephelus latifasciatus* | | NC_020784 |
| *Epinephelus fuscoguttatus* | | KP013758 |
| *Epinephelus bruneus* | | NC_013820 |
| *Epinephelus fuscoguttatus* × *Epinephelus lanceolatus* | | NC_028055 |
| *Epinephelus* *moara* | KP009977 | |
| *Epinephelus corallicola* | KP072053 | |
| *Epinephelus moara* × *Epinephelus lanceolatus* | KX147236 | |
| *Gymnocephalus cernua* | MK509809.1 | |
| *Sander vitreus* | KT211433.1 | |
| *Siniperca chuatsi* | JF972568.1 | |
| *Collichthys niveatus* | HM219223.1 | |
