# Supplementary figures and images for: rDNA and mtDNA analysis for the identification of genetic characters in the hybrid grouper derived from hybridization of Cromileptes altivelis (female) × Epinephelus lanceolatus (male)
Source: BMC Genom Data. 2024 Jan 12;25:5. doi: 10.1186/s12863-023-01188-5 (PMC10787421; doi:10.1186/s12863-023-01188-5)

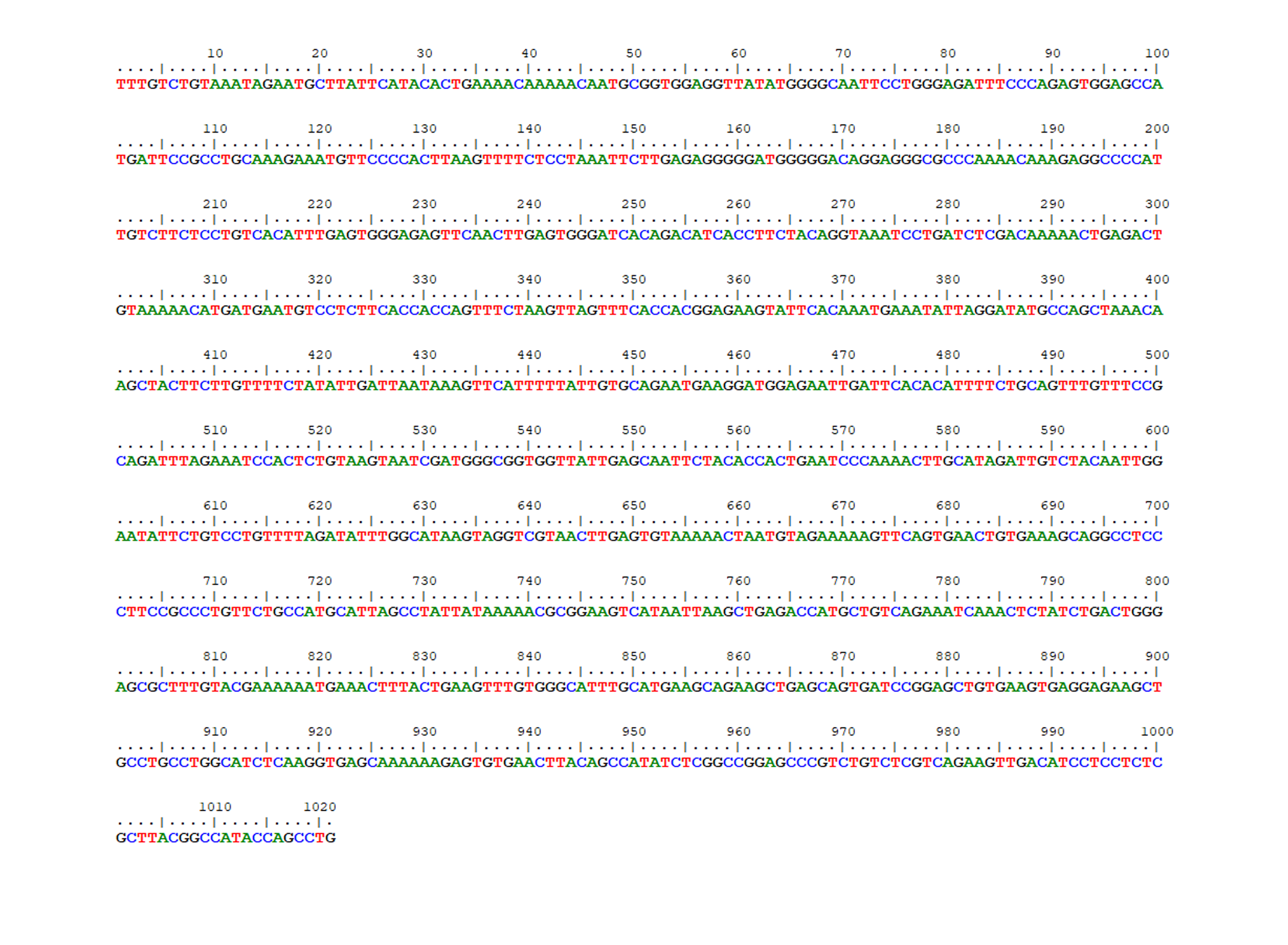

Supplement: Supplementary file 2 — Additional file 2: Supplementary Figure 1. Representative sequences of 5S rDNA intergenic spacers (IGS) sequence. [file 12863_2023_1188_MOESM2_ESM.tif]

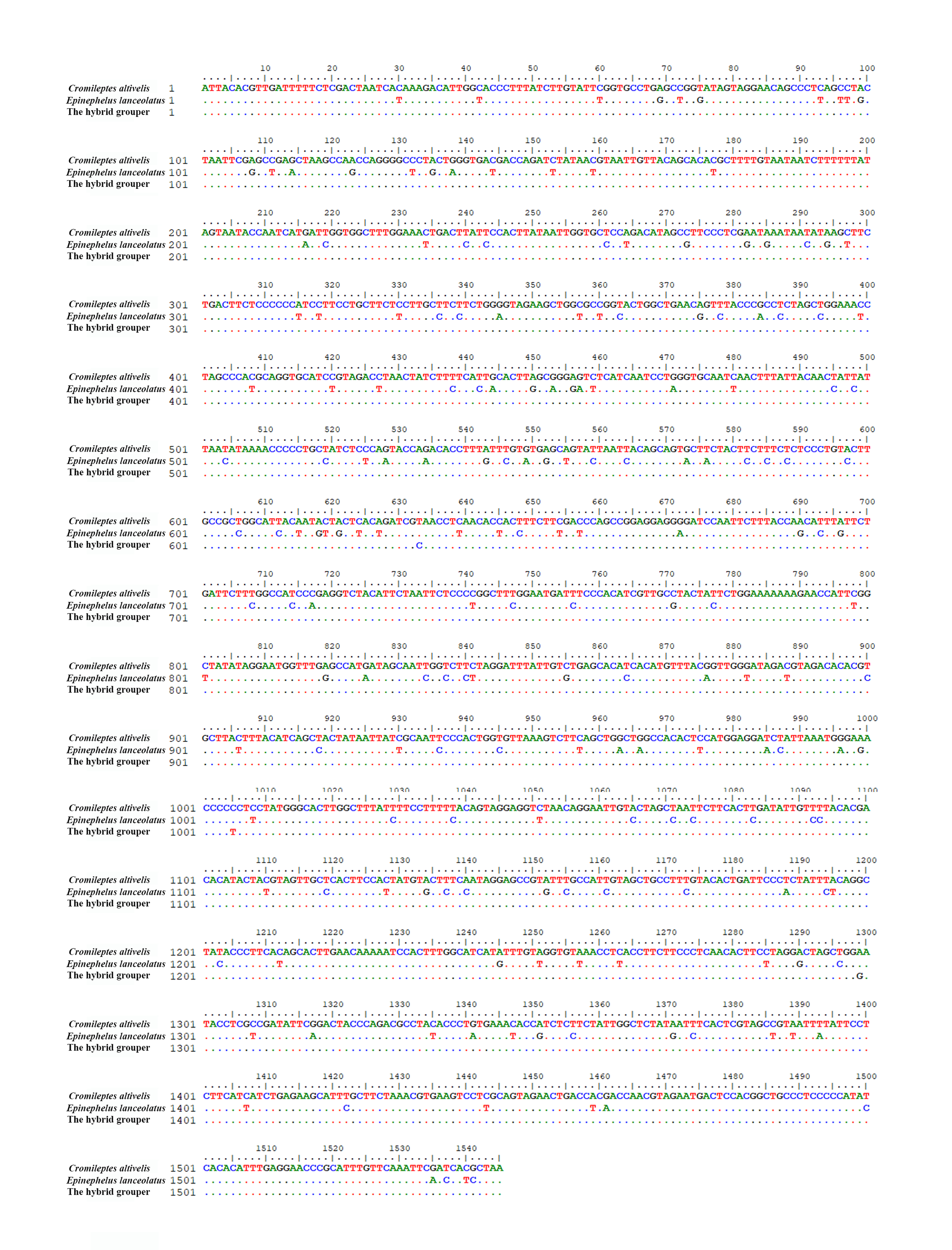

Supplement: Supplementary file 3 — Additional file 3: Supplementary Figure 2. Representative sequences of COI gene from Cromileptes altivelis, Epinephelus lanceolatus, the hybrid grouper. [file 12863_2023_1188_MOESM3_ESM.tif]

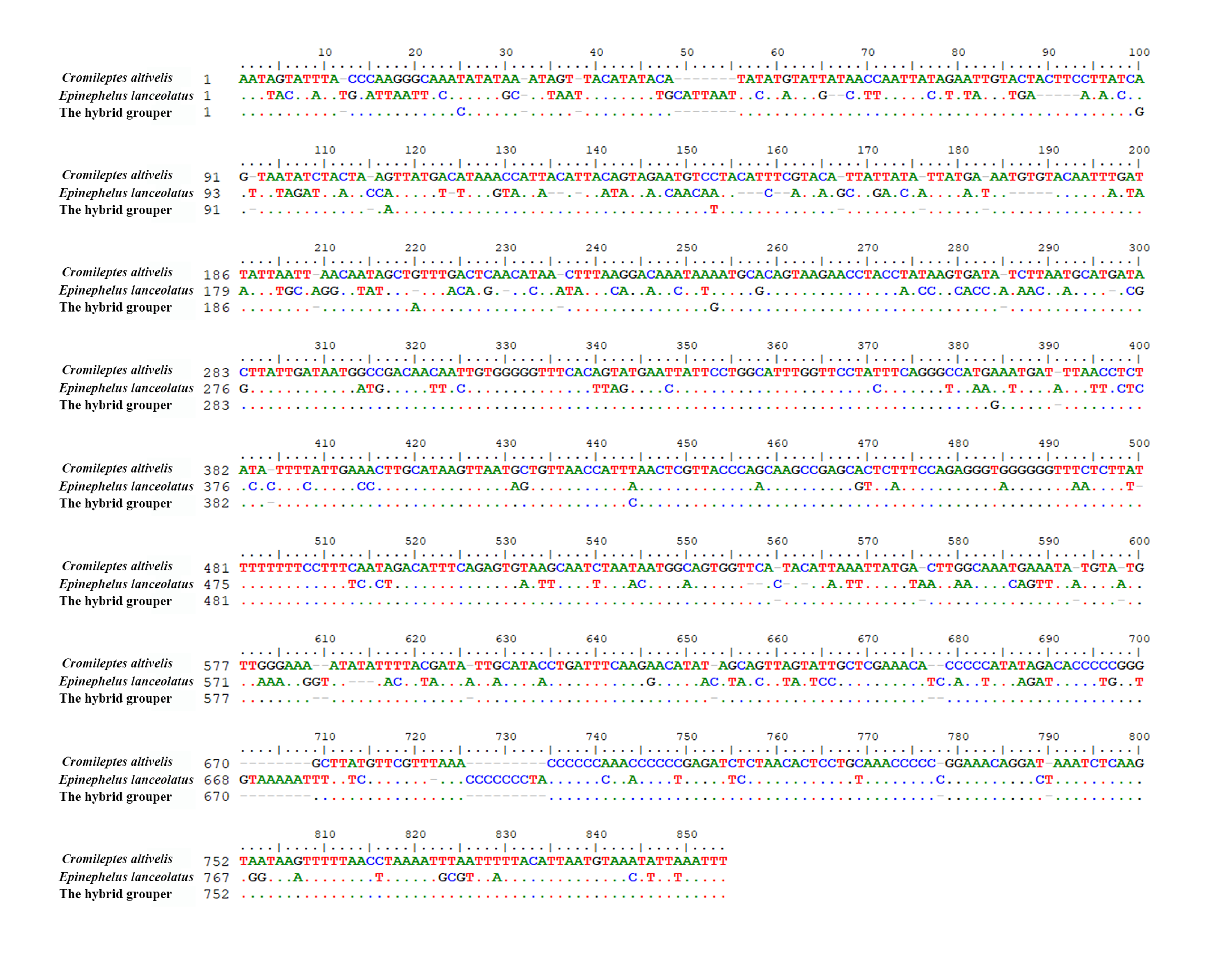

Supplement: Supplementary file 4 — Additional file 4: Supplementary Figure 3. Representative partial sequences of D-loop region from Cromileptes altivelis, Epinephelus lanceolatus, the hybrid grouper. [file 12863_2023_1188_MOESM4_ESM.tif]
